# Visual Illusions in Radiology: Untrue Perceptions in Medical Images and Their Implications for Diagnostic Accuracy

**DOI:** 10.3389/fnins.2021.629469

**Published:** 2021-06-11

**Authors:** Robert G. Alexander, Fahd Yazdanie, Stephen Waite, Zeshan A. Chaudhry, Srinivas Kolla, Stephen L. Macknik, Susana Martinez-Conde

**Affiliations:** ^1^Department of Ophthalmology, State University of New York Downstate Health Sciences University, Brooklyn, NY, United States; ^2^Department of Neurology, State University of New York Downstate Health Sciences University, Brooklyn, NY, United States; ^3^Department of Physiology and Pharmacology, State University of New York Downstate Health Sciences University, Brooklyn, NY, United States; ^4^Department of Radiology, State University of New York Downstate Health Sciences University, Brooklyn, NY, United States

**Keywords:** radiological error, illusions, false positives, perceptual expertise, image quality, medical image perception, medical images, false negatives

## Abstract

Errors in radiologic interpretation are largely the result of failures of perception. This remains true despite the increasing use of computer-aided detection and diagnosis. We surveyed the literature on visual illusions during the viewing of radiologic images. Misperception of anatomical structures is a potential cause of error that can lead to patient harm if disease is seen when none is present. However, visual illusions can also help enhance the ability of radiologists to detect and characterize abnormalities. Indeed, radiologists have learned to exploit certain perceptual biases in diagnostic findings and as training tools. We propose that further detailed study of radiologic illusions would help clarify the mechanisms underlying radiologic performance and provide additional heuristics to improve radiologist training and reduce medical error.

## Introduction

Most diagnostic errors in radiologic practice are secondary to failures of perception (Renfrew et al., 1992; Slater et al., 2006; Bruno et al., 2015; Waite et al., 2017, 2020). Some of these errors are driven by visual illusions that radiologists encounter as they analyze radiographs. Illusions are hard to rigorously define (Eagleman, 2001), and—while there have been efforts to precisely categorize or describe illusions (Ninio, 2014)—there is no consensus definition among perception scientists. In this paper, we define illusions as mismatches between physical reality and perception (see Westheimer, 2008; Murray and Herrmann, 2013; Shapiro and Todorovic, 2016 for similar approaches). In radiology, such mismatches can potentially interfere with accurate diagnosis.

Radiologists can fail to see pathologies due to perceptual biases—or see pathologies where none exist (Perrin and McBroom, 1987; Renfrew et al., 1992; Waite et al., 2019, 2020). Although missed diagnoses are more commonly discussed in the literature, false positives arising from over-diagnosing normal variations in anatomy as pathological can be harmful too, secondary to complications from unnecessary tests and treatments (Keats and Mark, 2001).

Although variable in the literature, the effective error rate in radiological studies is estimated to be about 4%, unchanged over the last 70 years (Waite et al., 2017). Worldwide, a 4% error rate would translate to approximately 40 million errors per year (Imanzadeh et al., 2020). Computer aided detection and machine learning methods promise to improve diagnostic accuracy, yet these same technologies place new demands on radiologists and can introduce novel sources of perceptual error (Slater et al., 2006; McGurk et al., 2008; Maskell, 2019).

Here, we review some of the more common illusions in radiology and their impact on clinical diagnosis (see Table 1). We discuss both diagnostic errors and the potential benefits of illusions. Understanding the differences between medical images and their perception in the observer can help enhance the ability of radiologists to detect pathology. Thus, radiologists armed with the knowledge of common illusions may not only better avoid misdiagnosis but even use illusions, when present, to help establish diagnosis (Buckle et al., 2013). In the future, error rates in radiology could be reduced through a better understanding of the role that illusions play, and radiology residents might be trained to both prevent and exploit such phenomena.

**TABLE 1:** Common visual illusions observed in radiology.

Type of illusion	Imaging modalities in which it is most commonly reported	Anatomical structures in which it is most commonly seen	Publications reporting this illusion in radiology
Mach bands	X-Ray	Musculoskeletal imaging; Chest x-rays, especially along the vertebral column	Lane et al., 1976; Daffner, 1977, 1989, 1995; Propper et al., 1980; Edholm, 1981; Berry, 1983; Daffner et al., 1986; Swann et al., 1987; Gordenne and Malchair, 1988; Papageorges and Sande, 1990; Papageorges, 1991; Cupples et al., 1996; Anbari and West, 1997; Chasen, 2001; Nielsen, 2001; Reeves, 2004; Thomson and Johnson, 2012; Buckle et al., 2013; Panikkath and Panikkath, 2014; Raby et al., 2014; Kattea and Lababede, 2015; Samei and Krupinski, 2018
Simultaneous contrast	CT	X-rays near skeletal structures, or CT with contrast material	Daffner, 1980, 1989; Gordenne and Malchair, 1988; Sogur et al., 2012
Pareidolia	CT	Brain	e.g., Griffin et al., 1998; Maria et al., 1999a,b; Hardy, 2003; Jacobs et al., 2003; Kato et al., 2003; McGraw, 2003; Gleeson et al., 2004; Graber and Staudinger, 2009; Lien et al., 2009; Maranhão-Filho and Vincent, 2009; Brancati et al., 2010; Roberts and Touma, 2011; Verma and Gupta, 2012; Foye et al., 2014; Sonam et al., 2014; Manley and Maertens, 2015; De Marzi et al., 2016; Massoud and Kalnins, 2016; Poretti et al., 2017; Shams et al., 2017; Ohmori et al., 2018; Ridley, 2018; Ridley et al., 2018
Parallax phenomena	X-Ray	No “most common” location identified in the literature	Volz and Martin, 1977; Daffner et al., 1982; Daffner, 1989; Thomson and Johnson, 2012; Buckle et al., 2013; Secgin et al., 2016

### Brightness and Contrast Illusions

Our brains do not detect the actual brightness of objects in the world, but instead compare an object’s physical luminance to that of nearby surfaces, frequently creating inaccurate representations of the natural world (Martinez-Conde and Macknik, 2017). Sometimes, the brightness and contrast illusions that result from such neural comparisons improve the visibility of structures on medical images, i.e., by enhancing boundary perception. Examples include any objects or surfaces where their physical luminance differs from their perceived brightness or contrast, such as Mach bands and simultaneous contrast effects.

#### Mach Bands

**FIGURE 1:**
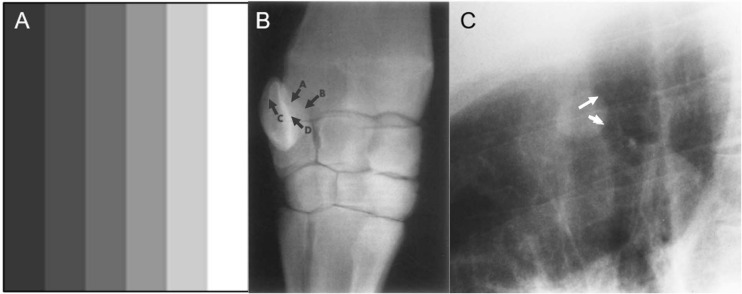
Examples of Mach bands. Classical Mach bands are apparent along the vertical edges of the stripes **(A)**. Although each individual stripe is physically uniform, its contrast intensity appears to differ between the left and the right edge, due to their respective proximity to adjacent bars with other luminances. Thus, at the border of any two adjacent bars, the edge of the lighter bar appears even lighter than in reality (a positive Mach band), while the edge of the darker bar appears even darker (a negative Mach band). **(B)** Radiograph of the carpus of a horse, in which some convex boundaries (arrows A,B), are associated with negative Mach bands, becoming almost imperceptible. Some concave boundaries (arrows C,D) are opaque and have prominent positive Mach bands. In this way, Mach bands provide shape cues: positive Mach bands may signal a concave shape, where negative Mach bands may signal a convex shape (from Papageorges, 1991). **(C)** A negative Mach band (arrows) helps define the nodule on this lateral radiograph (from Chasen, 2001).

Mach bands are a form of contrast enhancement, visible as a bandlike line at the edge of almost any shadow and at the borders between adjacent, overlapping objects with different luminance (see Figure 1A). They are commonly encountered in radiology on routine chest radiographs, in places where structures of different image intensities overlap (Lane et al., 1976; Daffner, 1977; Chasen, 2001), and occur most frequently along the vertebral column (Daffner, 1989; Raby et al., 2014). Mach bands can be “negative” (dark) or “positive” (bright), but only one type of Mach band is typically visible at each boundary created by most biological shapes (Edholm, 1981; Papageorges and Sande, 1990). Mach bands are often helpful in demarcating boundaries between anatomic structures—though this is not always the case. Moreover, negative Mach bands and their associated boundaries can be too dark to be seen clearly on radiographs.

Negative Mach bands are typically associated with convex (outward-curving) structures, and positive Mach bands with concave (inward-curving) structures (Papageorges, 1991). Papageorges (1991) suggested that these associations could be used to deduce the shape of unknown anatomical structures in radiographs, or to more accurately identify the shape of known structures (see Figure 1B). For instance, radiologists can use Mach bands to better visualize abnormalities that are present in radiographs, but obscured by overlapping structures: when one structure overlaps another, the resulting Mach band from the edge contrast difference can elucidate the shape and position of the occluded structure (Chasen, 2001; see Figure 1C). Information from Mach bands can be critical in cases where relying on memory to reconstruct the 3-dimensional anatomy would otherwise be difficult, overly complicated, or misleading.

**FIGURE 2:**
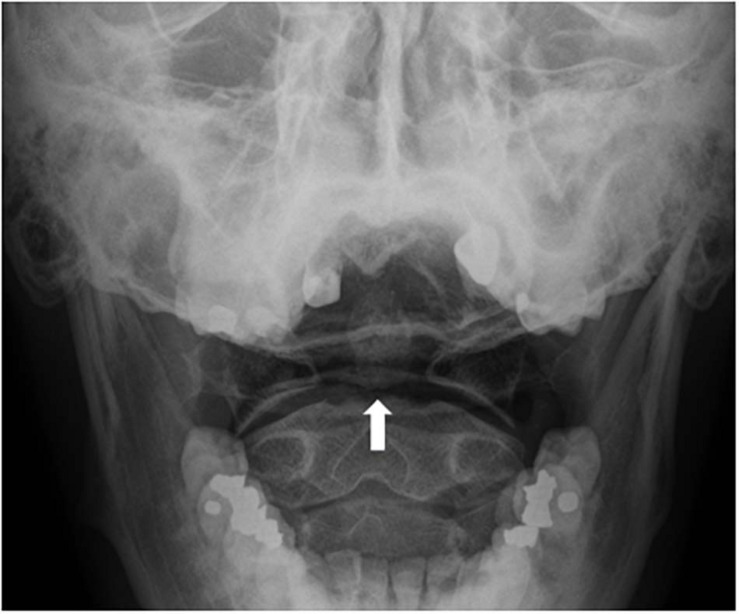
Mach bands across the base of the dens (a bone that projects from the spinal vertebra, also known as the “odontoid process”), can be mistaken for fractures (white arrow).

Unfortunately, Mach bands can also hinder accurate diagnosis: Mach bands that overlap with bone can be misperceived as fractures (Daffner, 1977, 1989; see Figure 2). Mach bands caused by skin folds can mimic the appearance of pneumothorax (air in the space between the thin pleural covering that surrounds the lungs; (Kattea and Lababede, 2015). Mach bands are also a cause of erroneous diagnosis of cavities (caries) on dental radiographs (Thomson and Johnson, 2012).

Indeed, trainees sometimes misinterpret Mach bands as fractures, only to be corrected by their mentors (see Reeves, 2004 for the description of one medical student’s experiences). Thus, residents are taught that when a Mach band might be present, they should look for additional findings that suggest a fracture: in the absence of such findings, apparent dark lines are likely indicative or Mach bands, rather than fractures (Samei and Krupinski, 2018). Expert radiologists are more adept at picking up on other subtle cues—or their absence, thereby avoiding diagnostic error (Nielsen, 2001). Thus, while the perceptual expertise of radiologists will not always prevent them from misperceiving images, prior knowledge and experience may improve diagnostic accuracy (Anbari and West, 1997). For example, in a case study reported by Panikkath and Panikkath (2014), Mach bands at the lateral margin of the right atrium were initially interpreted as evidence of pneumopericardium in a chest radiograph. Awareness that this might be a perceptual effect—and the discovery that other follow-up imaging did not show signs of air around the heart—revealed that this radiolucent shadow was in fact caused by Mach bands. The effects of context and experience on the interpretation of Mach bands in radiology have also been demonstrated experimentally by Nielsen (2001): dental students frequently misinterpreted a Mach band illusion as a root fracture, but experienced dentists (with three or more years of practice) only tended to provide the same misdiagnosis when they were given a correlative history, such as that the patient suffered trauma during a sporting event (a scenario in which a root fracture might be expected).

#### Simultaneous Contrast Effect

**FIGURE 3:**
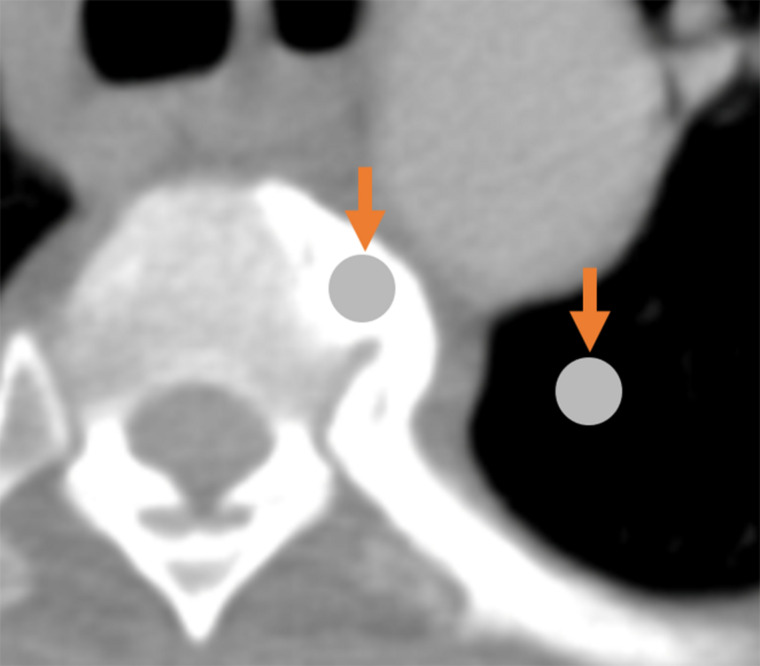
Demonstration of the simultaneous contrast effect: although the two circles indicated by arrows are physically identical, the circle on the left appears darker relative to the (seemingly-brighter) circle on the right.

The simultaneous contrast effect is another brightness/contrast illusion, occurring when differences in luminance between an object and its background, or between one object and another, alter the object’s perceived brightness. In radiological contexts, differences in background density can alter the perceived density of two adjacent objects due to simultaneous contrast (Gordenne and Malchair, 1988; see Figure 3). Importantly, whereas Mach bands usually cover a narrow area (resembling a thin band), simultaneous contrast can cover wide areas.

### Pareidolia

Pareidolia is the illusion of significance in meaningless sensory inputs. Everyday examples include seeing a face on the moon or finding animal shapes in clouds. We note that pareidolia does not always result in visual illusions and it can occur in other sensory domains: for example, hearing lyrics in music played backwards. Pareidolias result from the same neural processes that extract actual (rather than imagined) meaning from meaningful, real-world objects (Voss et al., 2011).

**FIGURE 4:**
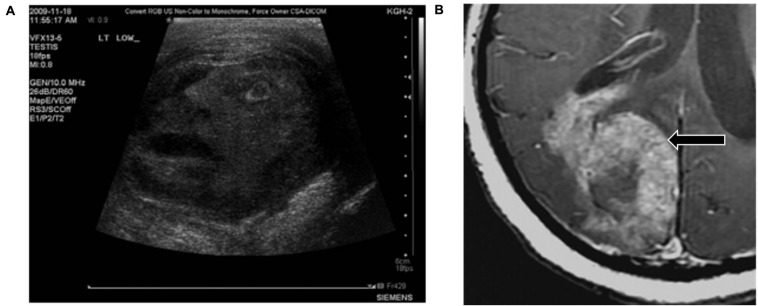
Examples of pareidolia that neither assist with nor hinder diagnosis. **(A)** The scrotal ultrasound from a patient with a testicular condition appears to resemble the face of a man in pain. **(B)** This lethal glioblastoma resembles a rabbit. Reproduced from Roberts and Touma (2011) and Massoud and Kalnins (2016), respectively.

Pareidolia often serves as an amusing finding that does not hinder or help the radiologist. For example, in one case report describing a man with painful inflammation on his testicles, the testicular mass on the ultrasound image resembled the face of a man in severe pain (Roberts and Touma, 2011; see Figure 4).

Yet, because radiologic diagnosis involves the recognition of patterns, pareidolias can be used in similar ways as other mental representations of normal and abnormal conditions exploited by expert radiologists. Importantly, because there is consistency in the perception of pareidolic elements across observers, mentors can share their own perceptual experiences with trainees, and highlight those pareidolia patterns that can aid the diagnostic process. Just as expert radiologists call to mind pre-existing mental representations (such as that of chronic lung disease) while attempting to fit cases to a possible diagnosis (Lesgold et al., 1988), pareidolia may help particular representations to be called to mind or fit to images.

Indeed, pareidolias can be representative of specific conditions, and therefore useful in diagnosis (Maranhão-Filho and Vincent, 2009). Radiologists have described hundreds of such diagnostic “signs”—visual analogies that suggest the presence of a condition or disease (Ridley, 2018; Ridley et al., 2018). Below, we list several pareidolias that serve as effective diagnostic heuristics.

#### The Snowman Sign

Radiologists often learn that the “snowman” sign in the pituitary region indicates that a pituitary macroadenoma is more likely than a meningioma. The characteristic “snowman” appearance of macroadenomas in that region—a “Figure 8” shape—results from the fact that macroadenomas are softer tumors that become indented where they pass through the sella turcica (the skull bone surrounding the pituitary gland) (Hess and Dillon, 2012).

#### The Swallow Tail Sign

In some cases, the *absence* of pareidolia can signal the presence of a disorder (De Marzi et al., 2016). For example, some linear or comma shapes (resembling the tail of a swallow) are present on normal images of the substantia nigra, but absent in most patients with Parkinson Disease or dementia with Lewy Bodies. Thus, “loss of the swallow tail sign” indicates likely Parkinson Disease or Lewy Body dementia (Shams et al., 2017).

#### The Molar Tooth Sign

**FIGURE 5:**
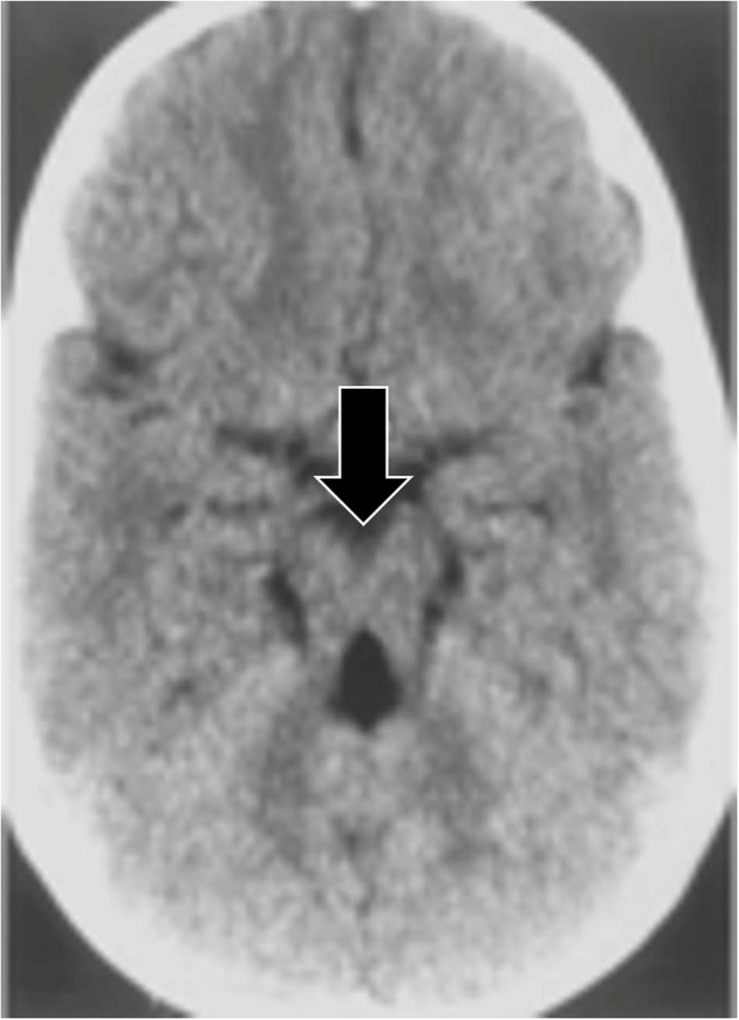
An axial CT image demonstrating the molar tooth sign, a pattern associated with Joubert syndrome. Lack of normal decussation of the fiber tracts of the superior cerebellar peduncles and the pyramids results in thickened and horizontally oriented superior cerebellar peduncles (McGraw, 2003; Romani et al., 2013). Along with the decreased anteroposterior dimension of the brainstem resulting from the absence of crossing fibers, and a deeper interpeduncular fossa (McGraw, 2003), these lead to the classic molar tooth appearance (from Gleeson et al., 2004).

In the “molar tooth sign,” the midbrain resembles a molar or wisdom tooth in axial CT scans (see Figure 5). The molar tooth sign was first observed in a rare condition known as Joubert syndrome, a ciliopathy (a disorder affecting cellular cilia) characterized by an abnormal respiratory pattern, ocular motor apraxia, hypotonia and developmental delay. The syndrome is genetically heterogenous with over 30 causative genes identified, and its characteristic morphology has been reported in 82–100% of Joubert Syndrome patients (Maria et al., 1999a; Poretti et al., 2017). The molar tooth sign is also consistently found in a variety of conditions that share similar features to classic Joubert Syndrome, but with varying causative genes and hence variable involvement of organ systems. Collectively these are referred to as Joubert Syndrome and Related Disorders (JSRD) (Manley and Maertens, 2015). The molar tooth sign is not typically observed on fetal MRI until the 22nd week of gestation, so further identification of the genetic factors causing JSRD could improve early detection (Fluss et al., 2006; Saleem and Zaki, 2010; Romani et al., 2013). In addition, JSRD patients consistently have hypoplasia of the cerebellar vermis, producing an abnormal cleft between the cerebellar hemispheres and another pareidolia, the “batwing appearance” of the fourth ventricle (McGraw, 2003).

#### The Hummingbird Sign

**FIGURE 6:**
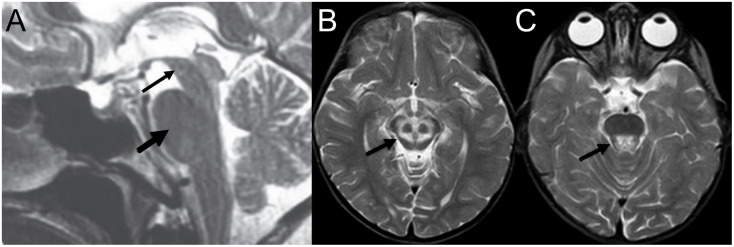
Examples of neuroradiological imaging pareidolia in central nervous system diseases. **(A)** Midbrain atrophy (thin arrow) without atrophy of the pons (thick arrow) results in the appearance of a hummingbird in patients with progressive supranuclear palsy. **(B)** Increased signal at the tegmentum with decreased intensity at the superior colliculi appears to represent a large panda head (arrow), and **(C)** a second head of a smaller panda is visible at the pons (**A** is modified from Verma and Gupta, 2012; **B,C** from Sonam et al., 2014).

Progressive supranuclear palsy (PSP), a degenerative disease characterized by ataxia and supranuclear vertical gaze palsy (Chen et al., 2010; Leigh and Zee, 2015; Alexander et al., 2018), is associated with the “hummingbird sign,” also called the “penguin sign” (Graber and Staudinger, 2009). On mid-sagittal plain MRI of PSP patients, midbrain atrophy appears to resemble a hummingbird (Kato et al., 2003; see Figure 6A). Because this midbrain atrophy is present only in PSP patients, the hummingbird sign can effectively differentiate PSP from Parkinson’s disease patients with a diagnostic sensitivity of around 100% (Verma and Gupta, 2012).

#### The Double Panda Sign

The “double panda sign” is associated with Wilson’s disease, characterized by copper accumulation in the body leading to psychiatric symptoms (Jacobs et al., 2003). It includes two separate panda faces: a “face of the giant panda” on the midbrain and a “face of the miniature panda” on the tegmentum region of the pons (see Figures 6B,C). Other disorders, such Methyl alcohol poisoning and Leigh disease, can also produce the double panda sign; thus, its presence does not result in a definitive diagnosis without additional findings (Das and Ray, 2006).

#### The Scottie Dog Sign

**FIGURE 7:**
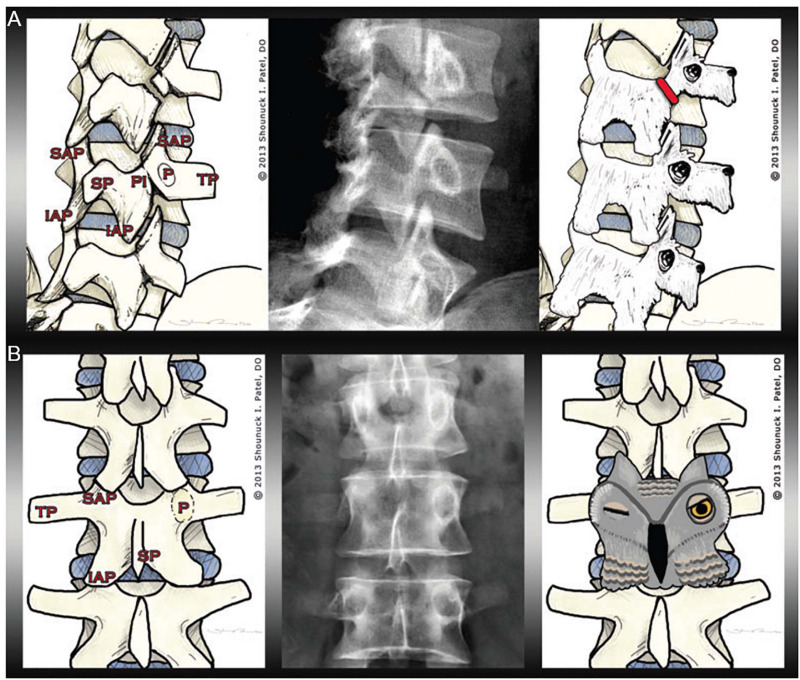
Examples of pareidolia within musculoskeletal pathology. The left image in **(A)** illustrates the anatomy of the lumbar spine, including the transverse process (TP), the superior articular process (SAP), the inferior articular process (IAP), the spinous process (SP), the pedicle (P), and the pars interarticularis (PI). The radiograph in the middle panel shows a fracture of the pars interarticularis. As demonstrated in the right panel, the vertebrae can be visualized as Scottie dogs, with pars interarticularis fractures resembling collars around the dogs’ necks. **(B)** The left image shows the musculoskeletal anatomy, including—crucially—the pedicle (P). In the middle panel, one pedicle is missing. The pedicles—as visualized in the right panel—resemble owl eyes: the owl appears to be winking when one pedicle is missing (for instance, if destroyed by metastatic cancer) (from Foye et al., 2014).

Pars interarticularis fractures are common sports injuries in young athletes (Syrmou et al., 2010). The “Scottie dog sign” helps radiology students to rapidly orient themselves to the different parts of the vertebrae, and then recognize this injury in oblique radiographs of the spine (Foye et al., 2014). Different parts of the vertebrae can be visualized as different parts of a dog. If the dog’s neck appears to have a collar or break, this represents a fracture or defect in the pars interarticularis (see Figure 7A).

#### The Winking Owl Sign

The “winking owl sign” is the most common finding in plain spinal x-rays in patients with symptomatic extradural metastasis (Livingston and Perrin, 1978). The cancer might not be recognized if the sign is not detected. Thus, the presence or absence of the “winking owl sign” sign can aid diagnosis, as the sign is not seen when the metastasis is intradural or extramedullary (Perrin et al., 1982). Foye et al. (2014) argued that teaching students the “winking owl sign” facilitates their detection of missing pedicles, allowing them to determine if any destruction is symmetrical (see Figure 7B).

The examples above indicate the value of pareidolia illusions as educational and training tools in radiology, easy for trainees to remember and apply quickly to improve diagnostic accuracy (Maranhão-Filho and Vincent, 2009; Foye et al., 2014; Manley and Maertens, 2015). Many radiologists use pareidolia in the practice of their profession, even if they are unfamiliar with the meaning of the term as an illusion involving pattern recognition (Maranhão-Filho and Vincent, 2009).

### Illusions Due to Viewpoint in Space

**FIGURE 8:**
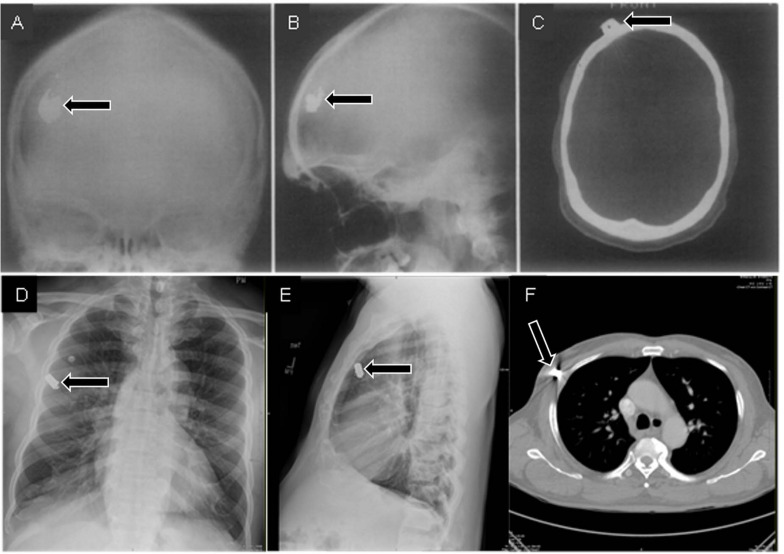
Frontal **(A)** and lateral **(B)** radiographs of a patient who suffered a gunshot wound to the skull seem to indicate that the bullet is within the skull. However, the skull does not appear to be fractured, and there is no indication that the bullet actually passed through the skull. A CT scan **(C)** provided the critical viewpoint to conclude that the bullet was just underneath the scalp (**A–C** from Daffner, 1989). In a separate case, frontal **(D)** and lateral **(E)** radiographs seem to indicate that a bullet is within the lung. However, a CT scan **(F)** revealed that the bullet was in the surrounding tissues.

When only 2D radiographic images are used, the limited viewpoints involved can prevent radiologists from seeing important anatomical structures. Except for cases in which contact between an object and local structures causes changes in opacity (thus providing a cue to the object’s relative location, called the “silhouette sign”; Kumaresh et al., 2015), it can be difficult or impossible to judge the anteroposterior location of an object from a single frontal image. In addition, the apparent position of structures can change with changes in line of sight, an effect called the “parallax phenomenon” (see Figures 8A–F).

**FIGURE 9:**
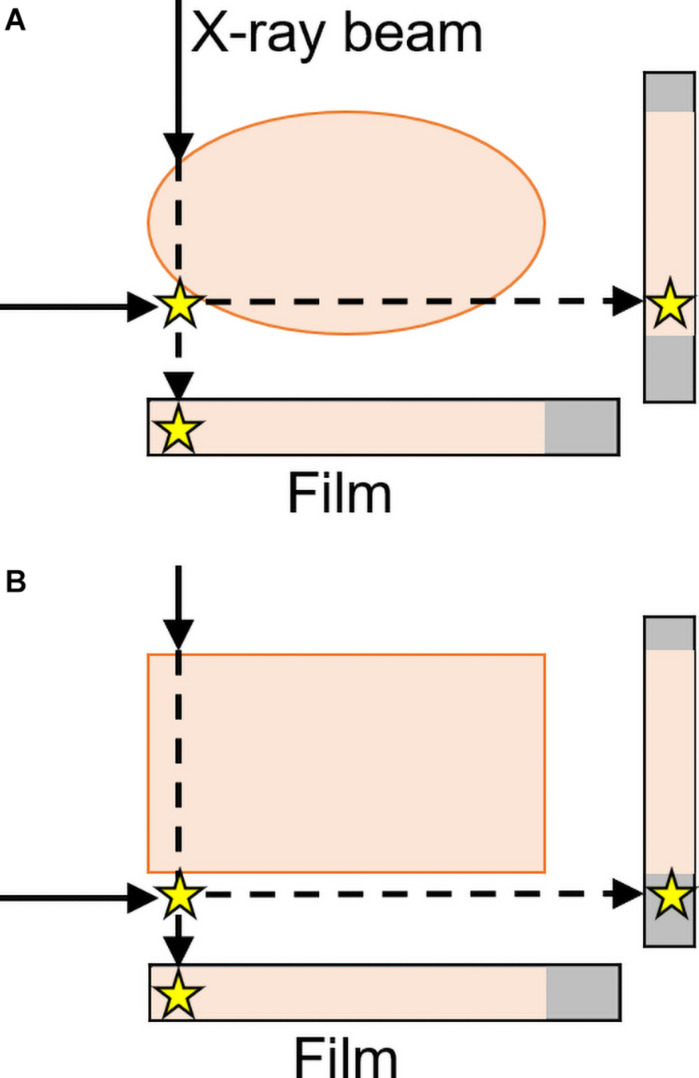
Frontal and lateral projections result in the star (representing an abnormality) either **(A)** appearing to be within the body or **(B)** correctly perceived as outside of the rectangular form with a lateral projection. Two views are thus sufficient to localize objects as either inside or outside a region, but only for rectangular parts of the body (Daffner et al., 1982). These “parallax phenomena” are both more likely and less easily resolvable with curved surfaces—like many structures in the body, including the skull and lungs in Figure 5—than with rectangular structures (inspired by an image from Daffner et al., 1982).

Illusions from parallax phenomena or overlap of structures can be resolved by taking additional images with oblique viewpoints (as opposed to only two 90° views) or by using different imaging methods (such as fluoroscopy) to view the structures from different angles as needed (Volz and Martin, 1977; Daffner et al., 1982; see Figure 9).

### Expectancy Effects

It is difficult to draw a hard line between pure vision vs. visual cognition: some biases typically considered to be cognitive can prevent observers from perceiving an image in a way that matches reality. For example, radiologists change the way they view images based on how likely they believe an abnormality will be—a cognitive phenomenon with important perceptual and diagnostic implications. When abnormalities are rare, as is typically the case in radiology, there can be a higher frequency of false-negative readings (Reed et al., 2011; Evans et al., 2013a; Wolfe et al., 2016). This “prevalence effect” also causes radiologists to increase the amount of viewing time for individual images when they expect the incidence of abnormalities to be high (i.e., when reading the chest radiographs of known smokers)—however, one study found that increased expectation of abnormalities was not linked to false positives (Evans et al., 2013a). Conversely, radiologists can fail to perceive unexpected findings even when seemingly obvious. In an illustration of the “inattentional blindness” phenomenon, radiologists may even look directly at something unexpected without perceiving it. In one study, a large image of a gorilla—48 times the size of a 5-mm lung nodule—was inserted into the last case examined during a nodule detection task. Twenty of twenty-four (83%) radiologists failed to perceive the gorilla, and the majority failed to notice it even after setting their eyes on it (Drew et al., 2013). Despite this high failure rate, expert radiologists performed better than ***non-experts***: every naïve observer tested failed to notice the gorilla. Knowing the patient history—and thus having some idea of what to look for—can additionally improve accuracy (McNeil et al., 1983; Berbaum et al., 1989, 1993): providing image readers with pertinent clinical history increased the accuracy of chest radiolograph interpretations from 16 to 72% for trainees and from 38 to 84% for experienced radiologists in one study (Brady et al., 2012).

### Distinguishing Illusion From Reality

Perception begins with the visual input itself; thus, medical image optimization can help prevent certain types of radiological illusions—especially those caused by artifacts of image formation (see Krupinski, 2006; Society for Imaging Informatics in Medicine, 2020). Light conditions, screen resolution, and room luminosity can all contribute to illusions. Thus, optimization of each of these factors can reduce uncertainty and decrease the prevalence of perceptual illusions (Sabih et al., 2011).

Radiologists have begun exploring the possibility of using smartphones for interpreting some radiographic images (Cruz et al., 2018), though some caution is advisable in these and similar approaches, as the incidence of radiologic illusions on (the smaller) smartphone displays is yet to be assessed. The development of new medical displays and techniques in radiology practice (i.e., those involving augmented or mixed reality) may moreover create new visual contexts for illusions to occur. For example, when head-mounted displays or operating equipment are augmented to overlay neuroradiological images on a patient during surgery, the surfaces, contours, and other visual attributes of the image may overlap with the patient’s form in ways that distort perception. Thus, it is critical to explore and be aware of potential illusions that may arise as a direct result of advances in medical technologies.

A judicious strategy to help distinguish reality from illusion is to rely on multiple sources of information for diagnosis, as opposed to a single finding. For example, the absence of secondary signs of trauma can help distinguish apparent from true fractures, where the presence of other findings can provide confirmatory evidence of a lesion. Similarly, second readings of uncertain radiographic findings (Sabih et al., 2011) are known to improve accuracy (Lauritzen et al., 2016). Thus, we recommend that referral clinicians look at their patients’ studies rather than relying solely on the radiology report. While radiologists’ perceptual abilities to detect abnormalities are more developed than those of referral clinicians (Reinus, 1995; Waite et al., 2019, 2020; Alexander et al., 2020), a second viewer who is more attuned to the patient’s history may notice details that the first viewer missed.

Ultimately, if a radiologist has difficulty distinguishing an illusion from a true lesion, repeating the image (ideally on a different axis) or using a different imaging modality could help. If needed, radiologists should rely on additional diagnostic tests and information from the patient history. If an image contradicts all other available evidence, a prudent radiologist should consider that the image interpretation may be wrong.

## Conclusion

Perceptual errors in radiology, including the illusions described above, are a significant contributor to patient harm (Waite et al., 2017, 2019). Yet, the perceptual training of radiologists relies on the informal teaching of some “tips and tricks” and “the techniques taught, while valid, do not result from a systematic review of the perceptual literature or understanding of the human eye-brain system” (Auffermann and Mazurowski, 2018, p. 472). Visual illusions can mimic lesions, causing radiologists to report pathology where there is none—leading to unnecessary workups or more invasive procedures. Thus, knowledge of how to detect and handle these illusions may help prevent premature or incorrect diagnoses.

Extending the existing knowledge about illusory perception from well-controlled lab studies to radiological practice is far from straightforward. Searching for abnormalities in radiologic images is likely to differ in many ways from searches conducted in non-radiological settings. Notably, radiologists can arrive to the correct diagnoses even when given very little time to search images and forced to guess (Evans et al., 2013b, 2016). However, prior research has found that radiologists are no better than non-specialists at finding hidden images in line drawings and “Where’s Waldo?” illustrations, suggesting that radiology training does not result in any cognitive or perceptual improvement that generalizes across search domains. Further, increased practice with “Where’s Waldo?” images does not enhance radiologic search accuracy (though it could improve overall search speed) (Sahraian et al., 2020). Instead, radiologic expertise is specific to radiologic images, suggesting critical differences between radiologic and non-radiological perceptual tasks (Nodine and Krupinski, 1998; see also Kelly et al., 2017).

Medical image perception has many qualities that are known to increase task difficulty in other contexts: the displays are complex and cluttered (Neider and Zelinsky, 2011), targets are unknown and vary widely in appearance (Alexander et al., 2019), are often low in salience (Biggs and Mitroff, 2015), are often similar to distractors (Alexander and Zelinsky, 2011, 2012), and are sometimes occluded by other structures (Alexander and Zelinsky, 2018). In addition, images may include more than one target or no targets (Clark et al., 2012; Waite et al., 2017). The intrinsic difficulty of the task may produce a set of behaviors that facilitate rapid and accurate performance in most cases but may increase the likelihood of particular illusions. A better understanding of how radiologists might be trained to avoid such illusions, and/or use them to their advantage, could enhance patient safety and save lives.

## Author Contributions

RA, FY, SW, SLM, and SM-C conceptualized, planned, and supervised the project. FY, RA, and SM-C reviewed the literature. FY, RA, SW, and SM-C wrote the main manuscript text. RA, FY, ZC, and SW prepared the figures and tables. All authors reviewed the manuscript.

## Conflict of Interest

The authors declare that the research was conducted in the absence of any commercial or financial relationships that could be construed as a potential conflict of interest.
